# Fourier-transform spectroscopy using an Er-doped fiber femtosecond laser by sweeping the pulse repetition rate

**DOI:** 10.1038/srep15726

**Published:** 2015-10-27

**Authors:** Keunwoo Lee, Joohyung Lee, Yoon-Soo Jang, Seongheum Han, Heesuk Jang, Young-Jin Kim, Seung-Woo Kim

**Affiliations:** 1 Department of Mechanical Engineering, Korea Advanced Institute of Science and Technology (KAIST), Science Town, Daejeon, 305-701, South Korea

## Abstract

Femtosecond lasers allow for simultaneous detection of multiple absorption lines of a specimen over a broad spectral range of infrared or visible light with a single spectroscopic measurement. Here, we present an 8-THz bandwidth, 0.5-GHz resolution scheme of Fourier-transform spectroscopy using an Er-doped fiber femtosecond laser. A resolving power of 1.6 × 10^4^ about a 1560-nm center wavelength is achieved by sweeping the pulse repetition rate of the light source on a fiber Mach-Zehnder interferometer configured to capture interferograms with a 0.02-fs temporal sampling accuracy through a well-stabilized 60-m unbalance arm length. A dual-servo mechanism is realized by combining a mechanical linear stage with an electro-optic modulator (EOM) within the fiber laser cavity, enabling stable sweeping control of the pulse repetition rate over a 1.0-MHz scan range with 0.4-Hz steps with reference to the Rb clock. Experimental results demonstrate that the P-branch lines of the H^13^CN reference cell can be observed with a signal-to-noise ratio reaching 350 for the most intense line.

## Introduction

Diverse femtosecond pulse lasers are available nowadays as broad spectral light sources suited for simultaneous spectroscopic detection of multiple absorption lines of a specimen^1^,^2^,^3^,^4^,^5^. Dispersive-type spectroscopy techniques can be adopted for such broad spectral sources, but special care is needed to prepare precision prisms or gratings that has to be tailored to cover the enlarged spectral bandwidth with a high resolving power^6^,^7^,^8^,^9^. On the other hand, Fourier-transform (FT) spectroscopy techniques enable flexible adjustment of the spectral bandwidth and the resolving power during the sampling process of the cross-correlation interferogram using a Michelson-type or equivalent two-arm interferometer^10^,^11^,^12^,^13^,^14^. Traditional FT spectroscopy techniques produce the interferogram mechanically by elongating the interferometer reference arm in scanning mode. Nonetheless, for the sake of high precision spectroscopy using femtosecond lasers, the reference arm scanning has to be performed with a sub-wavelength positioning accuracy over a long travel distance of up to several meters^14^. The burden of precise mechanical scanning can be relieved by combining a pair of femtosecond lasers of slightly different pulse repetition rates to form a multi-heterodyne light source^15^,^16^,^17^,^18^,^19^,^20^,^21^,^22^. This dual-comb FT spectroscopy technique permits acquisition of the interferogram by sampling the heterodyne beat-frequency signal down-converted to the radio-frequency regime. However, in order to achieve high precision spectroscopy without the frequency ambiguity or aliasing caused by the Nyquist sampling limit, both the combs have to be stabilized in synchronization to each other^20^, or the heterodyne beat signal has to be monitored by combining a set of continuous-wave lasers of known wavelengths^22^.

### Repetition rate sweeping FT spectroscopy

In this investigation, as an alternative method, FT spectroscopy is performed by sweeping the repetition rate (*f*_*r*_) of the femtosecond laser being used as the light source. This *f*_*r*_-sweeping method eliminates the interferometer arm scanning of conventional FT by employing a PZT micro-actuator so as to stretch the cavity length of the femtosecond laser^23^. In fact, the *f*_*r*_-sweeping method has already been successfully demonstrated not only for spectroscopy^24^ and lidar applications^25^ but also for long distance measurements^26^ and pulse duration estimation^27^,^28^. In principle, the interference overlap between pulses scales proportionally with the frequency range of *f*_*r*_-sweeping, but it is usually restrained to a few hundred Hz by the maximum elongation of the PZT micro-actuator. It is known that the interference overlap can be magnified significantly by providing a long unbalance length between the interferometer arms. However, such a long unbalance length is susceptible to the ambient temperature fluctuation which deteriorates the sampling accuracy and also the low signal-to-noise ratio in the resulting Fourier-transformed spectrum. Sampling errors may be corrected by incorporating a separate reference interferometer for elaborate tracing of the actual temporal variation of the unbalance length throughout the sampling process^23^. Such a posteriori error compensation requires excessive data recording and subsequently a large amount of computation to reconstruct the undistorted interferogram; besides these problems, quantifying the uncertainty of the utilized reference interferometer with traceability to a certified frequency standard remains an essential task. In our study, to cope with the problems of the previous attempts of *f*_*r*_-sweeping FT spectroscopy, special care was given to the real-time stabilization of the unbalance length by devising a dedicated length-monitoring interferometer. At the same time, the *f*_*r*_-sweeping was conducted by phase-locked control so that the FT sampling accuracy can be traceable to the Rb clock. In addition, emphasis was given to constructing a compact, robust FTS system demonstrating the effectiveness of femtosecond lasers as the light source for diverse spectroscopic applications over broad spectral bandwidths.

## Results

### Overall FT spectrometer system design

**Figure 1:**
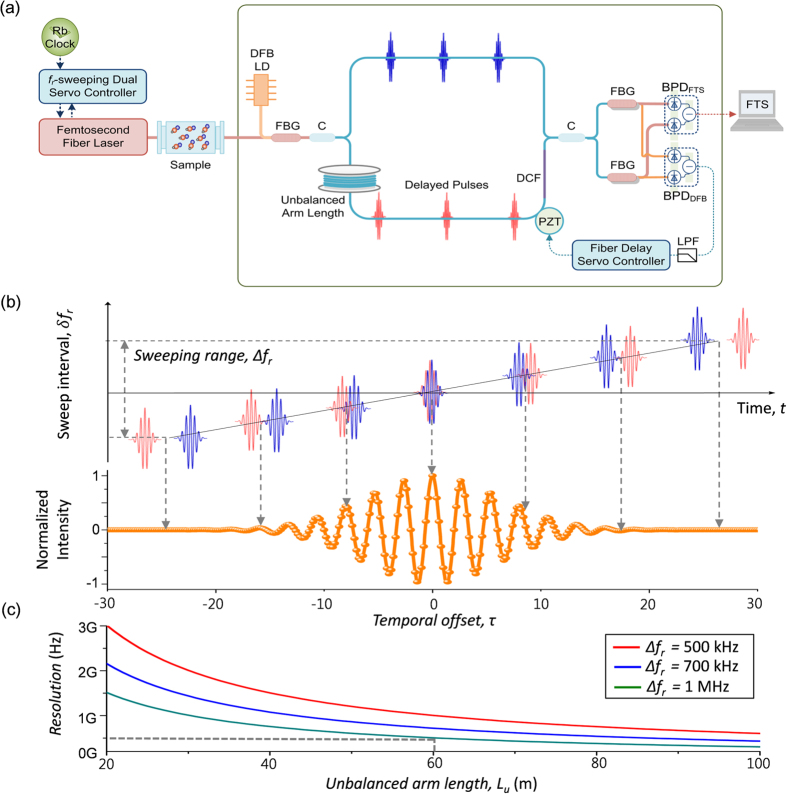
(**a**) The unbalanced-arm fiber interferometer for FT spectroscopy by *f*_*r*_-sweeping; *f*_*r*_: pulse repetition rate, DFB LD: distributed feedback laser diode, FBG: fiber Bragg grating, C: fiber coupler, DCF: dispersion-compensation fiber, BPD_FTS_: balanced photo-detector to monitor the interferogram of the femtosecond laser source, BPD_DFB_: balanced photo-detector to monitor the homodyne interference signal of DFB LD, LPF: low-pass filter. (**b**) Sampling of an interferogram with *f*_*r*_-sweeping. **(c)** Frequency resolution vs. unbalance arm length for various *f*_*r*_-sweeping ranges (Δ*f*_*r*_).

**Figure 2:**
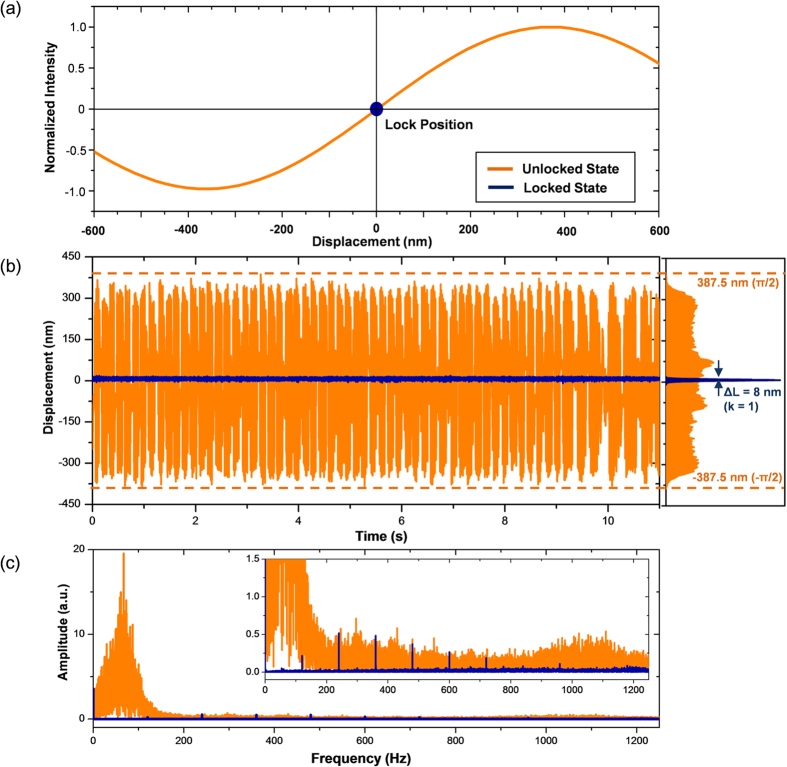
(**a**) Concept of active stabilization of the interferometer unbalance arm length by zero-phase locking of the embedded homodyne interferometer phase signal. (**b**) (Experiment) Zero-phase locking signals before and after stabilization. (**c**) Frequency distribution of the stabilized zero-phase locking signal with a magnified view (inset).

**Figure 3:**
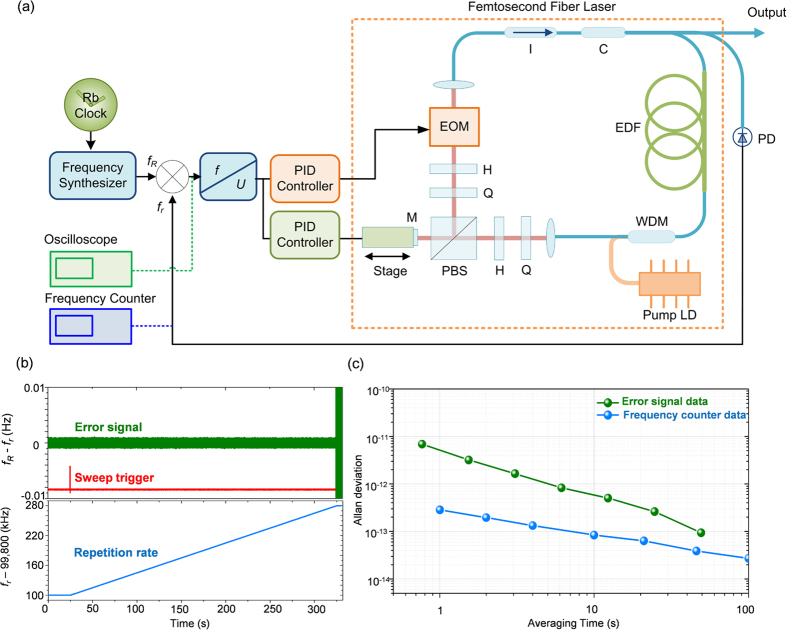
(**a**) System layout of dual-servo control for stable *f*_*r*_-sweeping. (**b**) Experimental results of *f*_*r*_-sweeping. The command *f*_*R*_ signal (blue) for *f*_*r*_-sweeping as initiated by the trigger signal (red) and the error signal of *f*_*R*_ − *f*_*r*_ (green) used as the control input. **(c)** Allan deviations of the *f*_*R*_ − *f*_*r*_ error signal (green) and the *f*_*r*_ signal (blue) measured from the frequency counter during *f*_*r*_-sweeping control.

**Figure 4:**
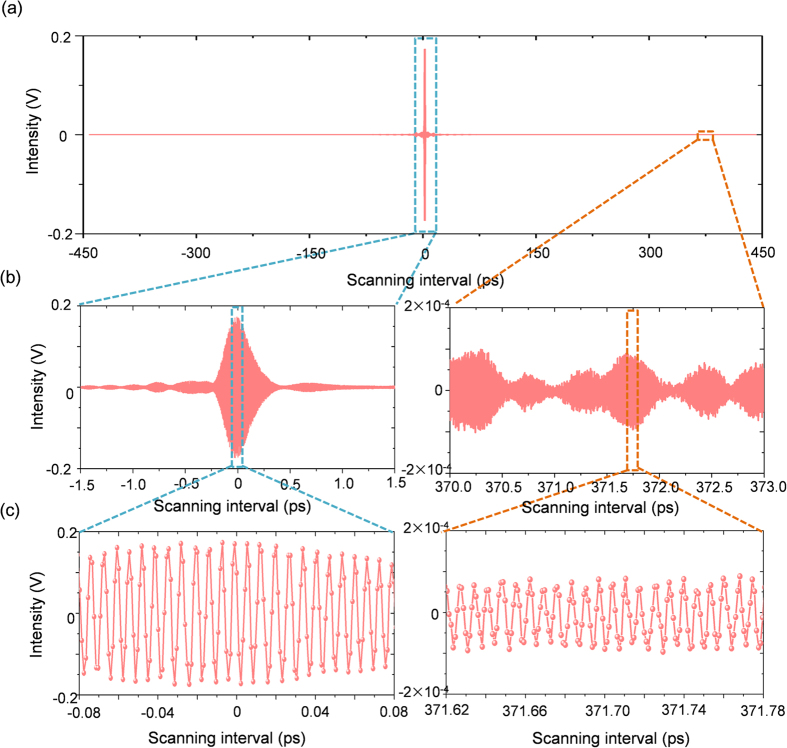
Interferogram measurement of the light source passing through the H^13^CN gas cell of 100-Torr pressure. (**a**) Overall interferogram obtained during *f*_*r*_-sweeping under PLL stabilization to the Rb clock. (**b**) Magnified views (X300) at two different optical delays. **(c)** Magnified views (X6000) showing the sinusoidal carrier signal resolved inside the pulse envelope.

**Figure 5:**
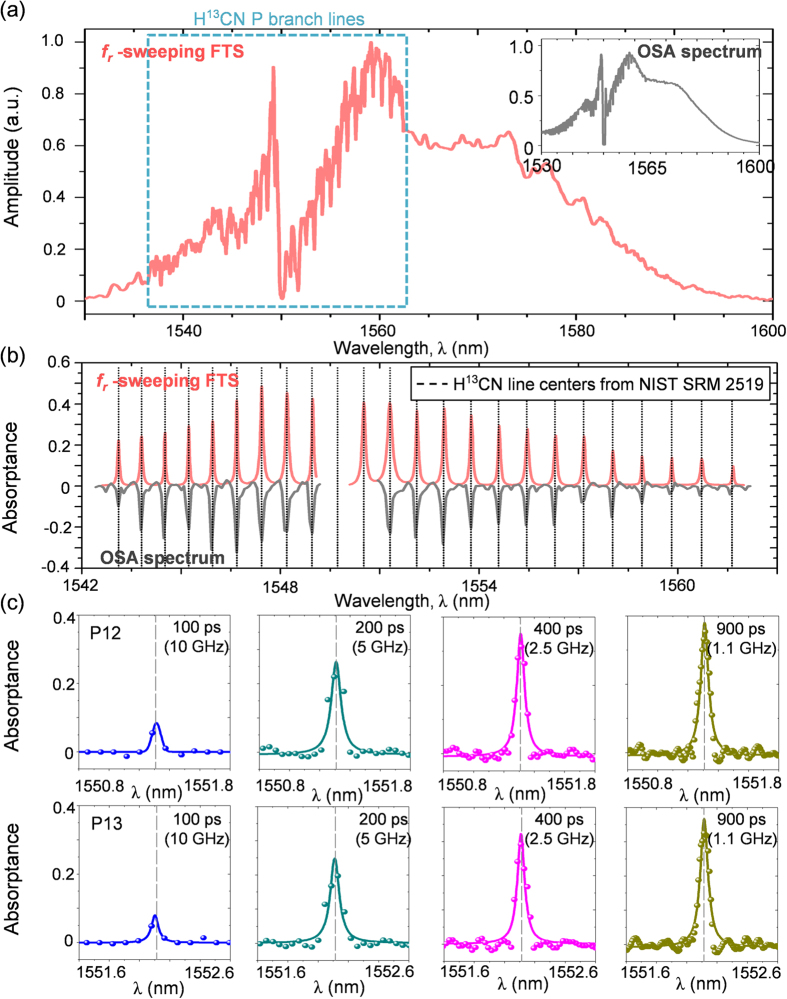
(**a**) Normalized absorption spectrum (red) of the H^13^CN cell of 100-Torr pressure (9-GHz absorption linewidth) measured by *f*_*r*_-sweeping FTS. For comparison, another spectrum (inset) is shown of the same H^13^CN cell measured using a commercial optical spectrum analyzer (OSA) of 8-GHz resolution. (**b**) Absorptance spectra of P-branch lines of the H^13^CN cell. **(c)** Magnified views of absorptance spectra for various sampling window widths (resolutions).

**Figure 6:**
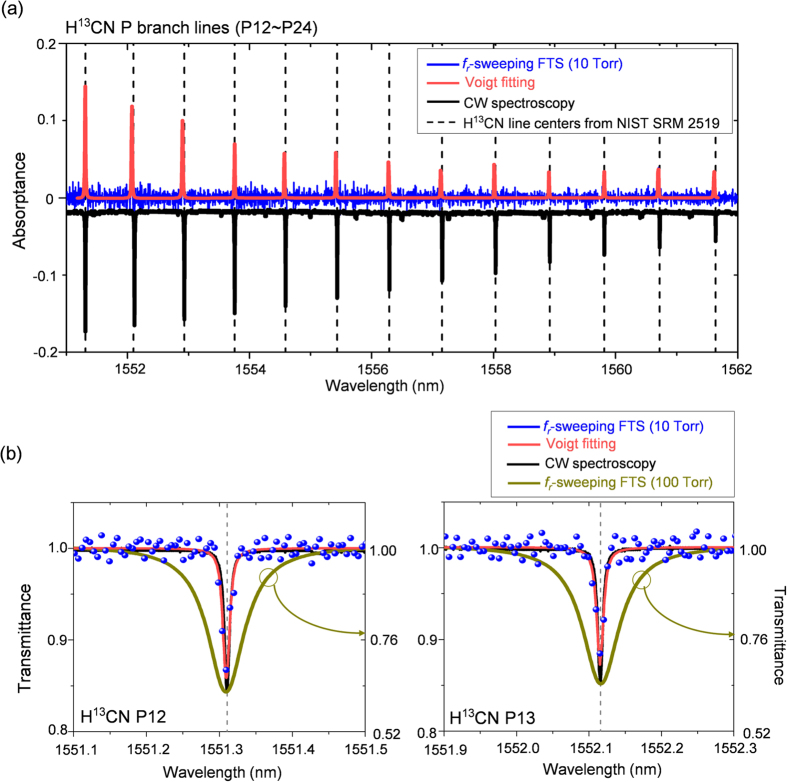
(**a**) Spectra of the H^13^CN cell of 10-Torr pressure (1.0-GHz absorption linewidth) measured using 0.5-GHz resolution *f*_*r*_-sweeping FTS and a tunable continuous-wave (CW) laser (3-MHz linewidth). Raw experimental data (blue dots) of the *f*_*r*_-sweeping FTS used for Voigt fitting are plotted in the background. (**b**) Voigt-fitted absorption lines of P12 & P13 of the 10-Torr H^13^CN cell. For comparison, the absorption lines (dark yellow) from Fig. 5c measured with 1.1-GHz resolution from the 100-Torr H^13^CN cell are reproduced.

For this study, we intended to demonstrate an enhanced *f*_*r*_-sweeping scheme of FT spectroscopy using an Er-doped fiber femtosecond laser. First, our FT spectrometer system was designed to detect interferograms by configuring a two-arm fiber interferometer of Mach-Zehnder type with a 60-m unbalance arm length (Fig. 1). Second, sampling errors caused by the ambient disturbance of temperature and vibration were minimized by stabilizing the unbalance arm length within a fluctuation level of ~8 nm by embedding a homodyne length-locking control scheme inside the fiber interferometer (Fig. 2). Third, the required *f*_*r*_-sweeping was accomplished over a 1.0-MHz repetition rate range in 0.4-Hz steps with reference to the Rb clock. To accomplish this, a dual-servo control method was devised by combining a linear mechanical micro-stage and an electro-optic modulator (EOM) within the laser cavity of the Er-doped fiber light source (Fig. 3). Fourth, it was verified that the consequent *f*_*r*_-sweeping capability permitted a 0.02-fs accuracy in acquiring interferograms over an optical delay of 900 ps (Fig. 4). Fifth, the proposed FT spectrometer system was tested by measuring the absorption lines of the hydrogen cyanide (H^13^CN) reference gas cell (Fig. 5). Finally, the performance of our spectrometer was evaluated by analyzing the measured the P-branch lines of the H^13^CN cell of 10-Torr (1.3 kPa) pressure over an 8-THz spectral bandwidth with a 0.5-GHz spectral resolution (Fig. 6).

### Interferometer design for *f*_*r*_-sweeping FT spectroscopy

Figure 1a shows the two-arm fiber interferometer designed in this investigation to attain the cross-correlation interferogram of the light source passing through the specimen. The light source is an Er-doped fiber laser made of a ring-type oscillator with a 3.0-m cavity length (*l*), which emits pulses of 120-fs duration at a nominal 100-MHz repetition rate (*f*_*r*_). A 60-m unbalance arm length (*L*_*u*_) is provided by inserting a fiber spool in one interferometer arm. The temporal offset (τ) between two pulses recombined at the interferometer exit is given as τ = *m*(1/*f*_*r*_) with *m* being an integer determined as *m* = *int*{*L*_*u*_/*l*}. For *L*_*u*_ = ~60 m and *l* = ~3.0 m, the integer *m* becomes 20. For an increment of δ*f*_*r*_ during *f*_*r*_-sweeping, the corresponding instantaneous variation of the temporal offset is given by δτ = −*m*(1/*f*_*r*_^2^)δ*f*_*r*_. This indicates that *m* acts as an amplification factor that allows for a large total offset (Δτ) between pulses for a given *f*_*r*_-sweeping range (Δ*f*_*r*_) in the sampling process (Fig. 1b). In FT spectroscopy, a large Δτ needs to be taken because the spectral resolution improves in proportion to 1/Δτ. Considering that the achievable maximum value of Δ*f*_*r*_ is about 1.0 MHz, the amplification factor *m* is set at 20, which extends Δτ to 2.0 ns with a resulting spectral resolution of 0.5 GHz (Fig. 1c). Finally, the interferogram is detected using a balanced photo-detector (BPD) which offers a high level of common noise subtraction.

### Unbalance arm length stabilization

Figure 2a shows how the unbalance arm length is stabilized by zero-locking of the interferometric phase generated from a continuous-wave DFB laser source which was installed through a fiber Bragg grating (FBG) to propagate together with the femtosecond laser source along the same interferometer arms (Fig. 1a). The DFB laser has a narrow 50-kHz linewidth about a 1550-nm center wavelength. The interferometric signal of the DFB laser is detected using a balanced photo-detector (BPD). For this process, a pair of fiber Bragg gratings (FBGs) of 100-GHz transmission linewidth about a 1550-nm center wavelength was used for filtering out the DFB laser. The fiber material of the unbalance arm length has a thermal expansion coefficient of 9.2 × 10^−6^ m/m °C, so the interferometric phase of the DFB laser undergoes a significant level of fluctuation arising from the ambient temperature change (Fig. 2b). For stabilization, a PZT-driven ring fiber bundle is inserted into the longer arm of the interferometer and controlled so that the interferometric phase is always locked to a zero-level phase reference (Fig. 2a). The zero-locking control bandwidth is 300 Hz, while the ambient disturbance mostly resides below 200 Hz (Fig. 2c). As a result, the interferometric phase is restrained at less than 0.01°, stabilizing the unbalance arm length within 8 nm (k = 1) (Fig. 2b). This stabilization level corresponds to the 0.02-fs temporal jitter in sampling the interferogram.

### Dual-servo control for *f*_*r*_-sweeping over a wide dynamic range

The process of *f*_*r*_-sweeping demanded in our FT spectroscopy was accomplished using a dual-servo mechanism to control the ring-fiber cavity length of the laser source (Fig. 3a). The dual-servo mechanism was devised by combining a mechanical stage of linear motion with an electro-optic modulator (EOM). The linear stage was driven by a stepping motor that provides coarse control over a long travel distance of tens of millimeters while moving the end position of the reflection mirror (M) installed inside the laser cavity. At the same time, fine control was achieved by the EOM which is made of a 40-mm thick MgO:LiNbO_3_ crystal to yield a 1.5-μm cavity elongation for a 200-V input, which induces a 40-Hz variation in *f*_*r*_-sweeping. The ramp-type command signal (*f*_*R*_) for *f*_*r*_-sweeping was generated using a frequency synthesizer that is synchronized to the Rb clock (2 × 10^−11^ stability @ 1 s). At the same time, the error signal (*f*_*R*_ − *f*_*r*_) was monitored in real time using a frequency counter along with a digital oscilloscope; this signal was subsequently fed into the dual-servo control system to regulate both the linear stage and the EOM simultaneously with control bandwidths of 1 kHz and 1 MHz, respectively. When *f*_*r*_-sweeping was conducted over a 180-kHz range during a 300-s time period, the error signal remained in the sub-mHz range (Fig. 3b) with a 5 × 10^−12^ stability @ 1 s (Fig. 3c). This experimental result proves that our dual-servo mechanism is capable of providing 0.5-mHz jitter (k = 1) in the sweeping process with direct traceability to the Rb clock. The *f*_*r*_-sweeping speed currently remains at a level of 600 Hz/s; it is limited by the Rb-clock-referenced signal generation speed of the frequency synthesizer used in our experiments even though the EOM is capable of responding to the command signal at 1.0-MHz bandwidth^29^. The *f*_*r*_-sweeping will speed up when the bottlenecking frequency synthesizer is replaced with a faster, custom-made electronic device later on.

### Interferogram sampling

The performance of our FT spectroscopy system was evaluated by observing the P lines of the H^13^CN gas corresponding to 2ν_3_ rotational-vibrational absorptions. Two different cells were used: one had an internal gas pressure of 100 Torr (13.3 kPa) and the other had an internal pressure of 10 Torr (1.33 kPa); both cells had the same length of 165 mm. Figure 4 shows an interferogram obtained using the 100-Torr cell, of which the absorption linewidth is 9-GHz in terms of full-width at half-maximum (FWHM)^30^,^31^. The requisite *f*_*r*_-sweeping was provided over a range of 99.907–100.373 MHz with a step size of 0.4 Hz, i.e. Δ*f*_*r*_ = 466 kHz and δ*f*_*r*_ = 0.4 Hz, following a ramp-input *f*_*R*_ command signal synthesized during a time period of 2,900 s. The cavity length underwent a 14-mm elongation. If the unbalance arm was set to zero, the cavity length extension should have been as long as 265 mm. The temporal sampling resolution was 0.76 fs over a temporal window size of 883 ps (Fig. 4a). The X300 magnified view (Fig. 4b) reveals that the cross-correlation interferogram between pulses appears to be nonsymmetrical. This is because the pulses passing through the long unbalance arm undergo a ~250-fs group delay due to dispersion, while the pulses of the short arm experience no significant dispersion. The X6000 magnified view (Fig. 4c) clearly shows a sinusoidal variation of the fundamental 1560-nm wavelength carrier signal seen underneath the interference envelop. The temporal sampling uncertainty is estimated to be 0.02 fs—one order of magnitude less than the 0.76-fs sampling resolution – being mainly influenced by the 8-nm thermal fluctuation of the unbalance arm length occurring during *f*_*r*_-sweeping (See Methods). Such high sampling accuracy reduces the noise level of the Fourier-transformed spectrum so that the absorption lines of the specimen can be detected with better signal-to-noise ratio and consequently less uncertainty in their absolute frequency positions.

### FT spectrum measurement

The sampled double-sided interferogram was processed by apodization with a Hanning function; it was then Fourier-transformed to obtain absorption spectra. No additional post-processing was necessary to compensate for the sampling error. An exemplary measured spectrum is shown in Fig. 5. The absorptance of the spectrum is calculated using the formula of *A*_*λ*_ = 1 − (*I*_*1*_/*I*_*0*_). *I*_*1*_ is the intensity of the radiation transmitted through the sample material; *I*_*0*_ is the total intensity of the incident radiation. The spectrum shown in Fig. 5a clearly reveals the absorption lines of the H^13^CN cell (100-Torr pressure) over a spectral bandwidth of ~8 THz spanning the wavelength range of 1530–1600 nm. The spectral distortion appearing near 1550 nm is due to a Kelly side peak which may be suppressed by dispersion control of the oscillator even though the effect of the peak on the spectrum measurement is minimal. The achieved frequency resolution is estimated to be 1.1 GHz, equivalent to 0.01 nm in wavelength, which is sufficient to resolve absorption peaks of 9-GHz linewidth^31^ as shown in Fig. 5b. The P-branch H^13^CN line positions were fitted by Lorentzian profile (Methods). For comparison, a raw spectrum obtained from the same H^13^CN cell using a commercial optical spectrum analyzer (OSA) made of a diffraction grating of 8 GHz resolution is included in Fig. 5b. It should also be noted that when the time window size is reduced from 900 ps to 100 ps (Fig. 5c) by restricting the temporal offset (Δτ) in sampling, the measured absorption profiles become shallower in depth and more dispersed in width. This consequently weakens the resolving power, deteriorating the system’s ability to accurately identify the absolute peak positions.

### 0.5-GHz resolution FT spectroscopy

Figure 6 shows another set of experimental result in which the absorption lines of the 10-Torr H^13^CN cell (typical linewidth: 1.0 GHz) were measured with enhanced resolving power. For this experiment, *f*_*r*_-sweeping was conducted over a range of 1.05 MHz with a step size of 0.4 Hz so as to achieve an enlarged window size of 2.0 ns. The temporal sampling resolution was maintained at 0.76 fs as before. The time consumed to synthesize the ramp-input command *f*_*R*_ signal in synchronization with the Rb clock was 6,200 s. As results, absorption lines were observed over the same spectral range of ~8 THz that was used in the previous experiment; however, frequency resolution was enhanced to 0.5 GHz. Voigt-fitted P12 and P13 absorption lines (Fig. 6b) were clearly resolved their 1.0-GHz linewidth profiles, which was not possible in the previous experiment when the frequency resolution was set at 1.1 GHz (Fig. 5). The enhanced resolution also allowed for improved identification of line peaks, Voigt-fitted line centers had discrepancies of 35 MHz (k = 1) from their certified absolute positions^30^. The signal-to-noise ratio was found to be 350 for the most intense line. This result was compared to the case achieved using a continuous-wave laser (Fig. 6b) which offered approximately 12% higher peak amplitudes with 3-MHz resolution.

## Discussion

Our *f*_*r*_-sweeping experimental work demonstrates 8-THz bandwidth, 0.5-GHz resolution FT spectroscopy, which offers a 1.6 × 10^4^ resolving power about a 1560-nm center wavelength. The line center discrepancy between the Voigt-fitted P-branch line positions of the H^13^CN reference cell and their certified frequency positions is ~35 MHz. This identification accuracy of absolute peak positions is attributable to the temporal sampling uncertainty of 0.02 fs achieved by stabilization of the unbalance arm length as well as continuous real-time referencing of the repetition rate to the Rb clock during *f*_*r*_-sweeping. It is anticipated that the proposed scheme of *f*_*r*_-sweeping FT spectroscopy can be exploited for diverse applications particularly for simultaneous observation of multiple chemical components of air-pollution in the atmosphere.

## Methods

### Temporal sampling accuracy.

In our FT spectroscopy experiment, the temporal sampling accuracy is affected dominantly by two stability factors related to the pulse repetition rate (*f*_*r*_) and the unbalance arm length (*L*_*u*_). First, dual-servo control of the *f*_*r*_-sweeping (Fig. 3) was found to provide a 0.5-mHz jitter level (k = 1) in the instantaneous value of *f*_*r*_ throughout the sweeping process, leading to a 0.0002 fs contribution to the sampling accuracy. Second, zero-locking stabilization control of the homodyne interferometric phase (Fig. 2) was performed within an 8-nm jitter level (k = 1) for the given *L*_*u*_ of 60 m; this resulted in a 0.02-fs level of sampling accuracy. Combining the two stability factors, the standard deviation of the temporal sampling accuracy is estimated to be σ_τ_ = [(0.0002 fs)^2^ + (0.02 fs)^2^]^1/2^ = 0.02 fs.

### Absorption line fitting

For the 10-Torr H^13^CN cell used in our experiment, a Voigt profile was found more suitable to fit absorption line shapes since the line broadening is known to be affected by both the intermolecular collision of a Lorentzian profile and the Doppler effect of a Gaussian profile. The Voigt function used in our fitting is; y = A{2ln(2)Γ_L_/(π^1.5^Γ_G_^2^)}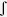 exp(−t^2^)/[{sqrt(ln2)(Γ_L_/Γ_G_)}^2^ + {sqrt(4ln2)(ω − ω_0_)/Γ_G_ − t}^2^] dt where A is the amplitude, Γ_L_ the full width at half maximum (FWHM) of the Lorentzian function, Γ_G_ the FWHM of the Gaussian function, ω the angular frequency, and ω_0_ the resonance angular frequency^20^,^30^. On the other hand, for the 100-Torr HCN cell of which the line broadening is dominated by intermolecular collisions, absorption lines were fitted by the Lorentzian function of y = (A/π)[Γ/{(ω − ω_0_)^2^ + Γ^2^}] where A is the amplitude, Γ is the full width at half maximum (FWHM), ω is the angular frequency, and ω_0_ is the resonance angular frequency^20^,^30^.

## Additional Information

**How to cite this article**: Lee, K. *et al.* Fourier-transform spectroscopy using an Er-doped fiber femtosecond laser by sweeping the pulse repetition rate. *Sci. Rep.* **5**, 15726; doi: 10.1038/srep15726 (2015).
